# Supplementary material for: Condemnation of Porcine Carcasses: A Two-Year Long Survey in an Italian High-Throughput Slaughterhouse
Source: Vet Sci. 2023 Jul 24;10(7):482. doi: 10.3390/vetsci10070482 (PMC10386549; doi:10.3390/vetsci10070482)
Supplement: Supplementary file 1 [file vetsci-10-00482-s001.zip › vetsci-2511444-supplementary.pdf]

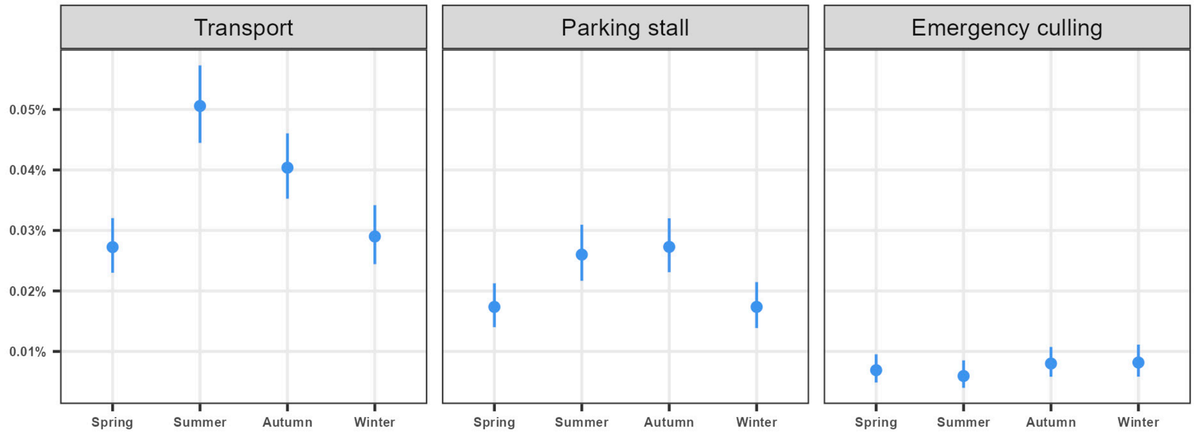

Figure S1.

Proportions with exact confidence interval about pigs unfit for slaughtering. Meteorological season were extracted from date of slaughtering and defined as follows: spring from March 1st to May 31, summer from June 1st to August 31st, autumn from September 1st to November 30st, winter from December 1st to February 28st.

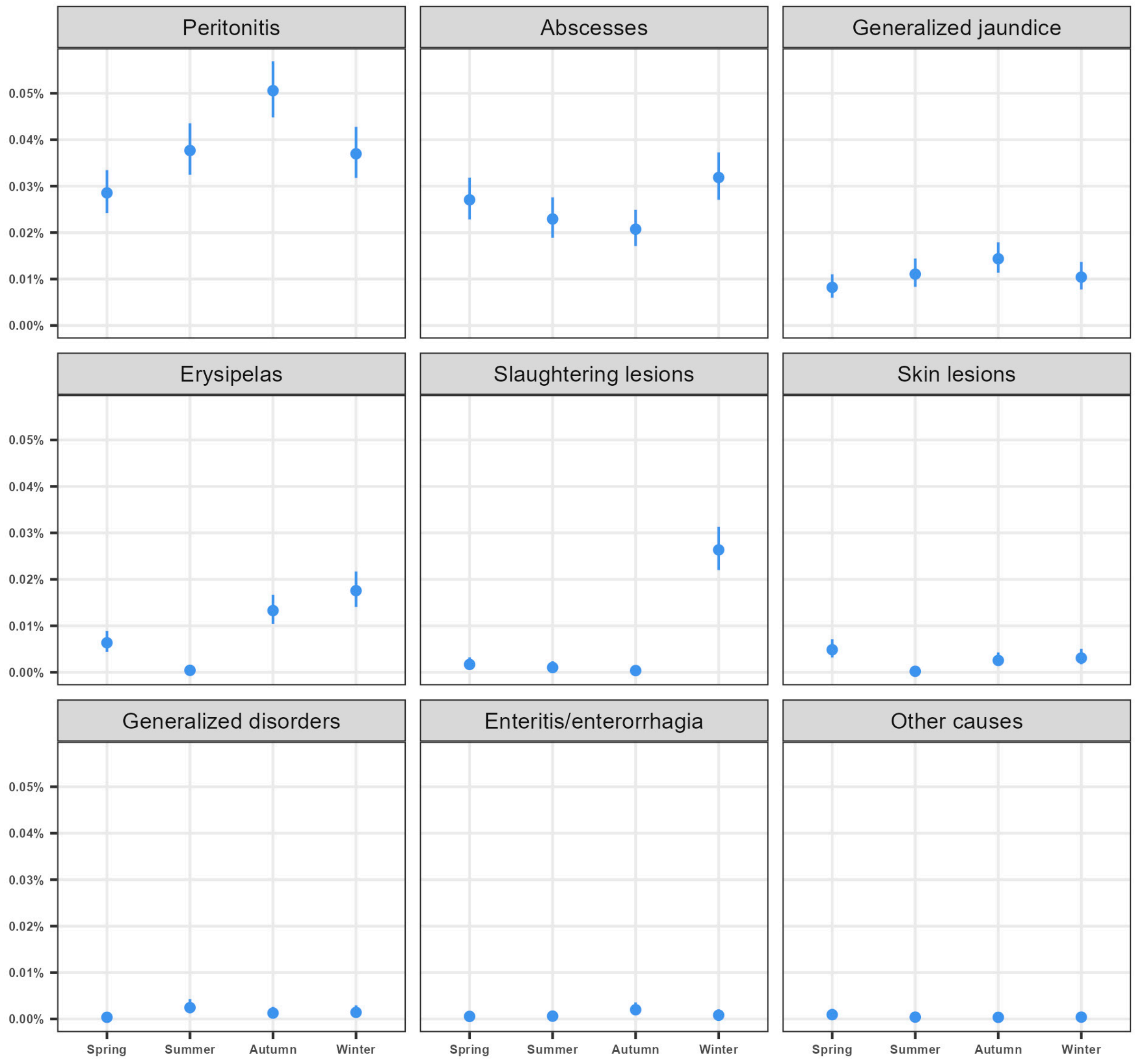

Figure S2.

Proportions with exact confidence interval about post-mortem carcass condemnations. Meteorological season were extracted from date of slaughtering and defined as follows: spring from March 1st to May 31, summer from June 1st to August 31st, autumn from September 1st to November 30st, winter from December 1st to February 28st.
